# Supplementary material for: In vitro analysis of Fusobacterium Polymorphum from oral leukoplakia patients identifies “high-risk” isolates
Source: bioRxiv. 2025 Oct 1:2025.10.01.679755. Preprint. [Version 1] doi: 10.1101/2025.10.01.679755 (PMC12621699; doi:10.1101/2025.10.01.679755)
Supplement: Supplement 1 [file media-1.docx]

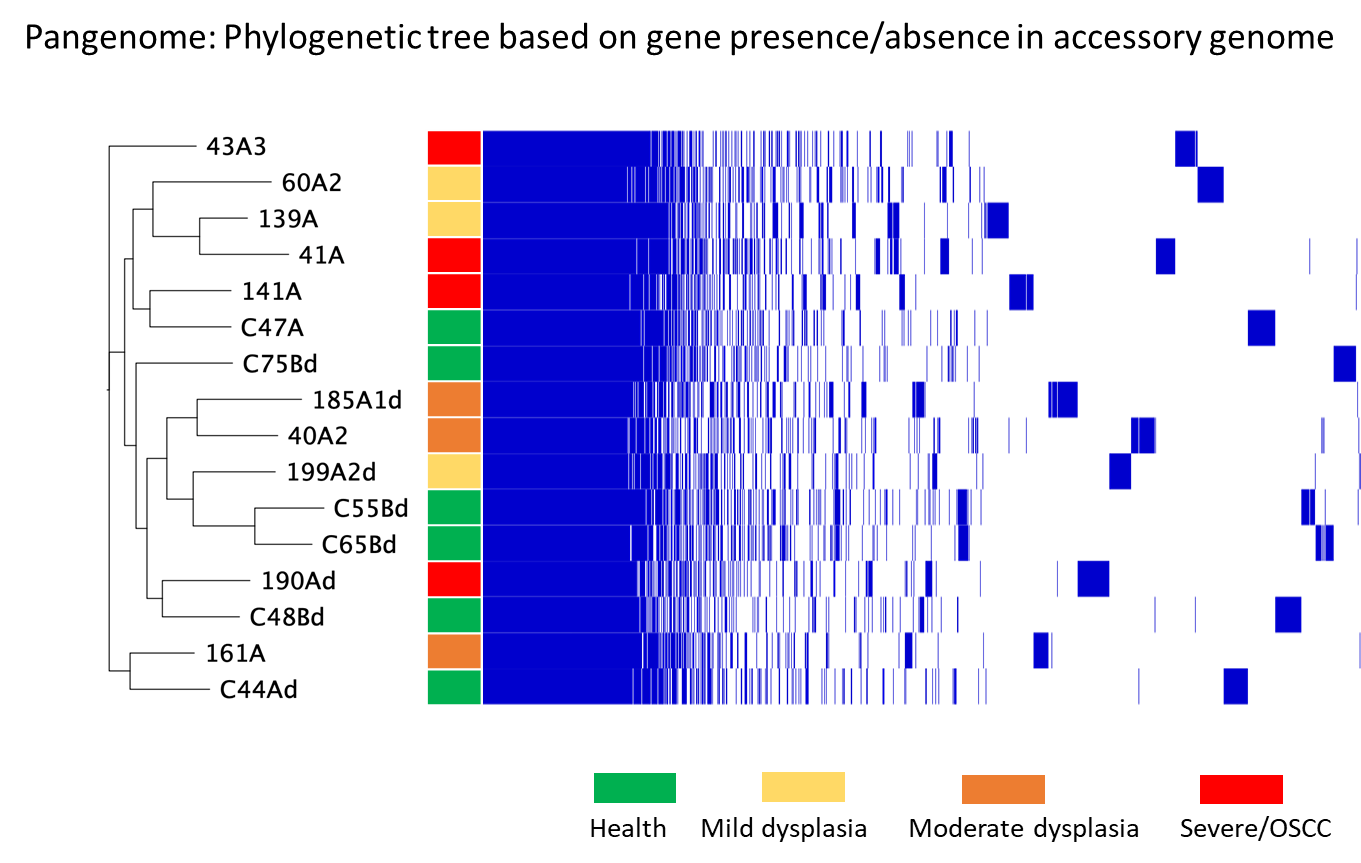


**Supplementary Figure 1. Accessory pangenome analysis.** Phylogenetic tree based on gene presence/absence in accessory genome


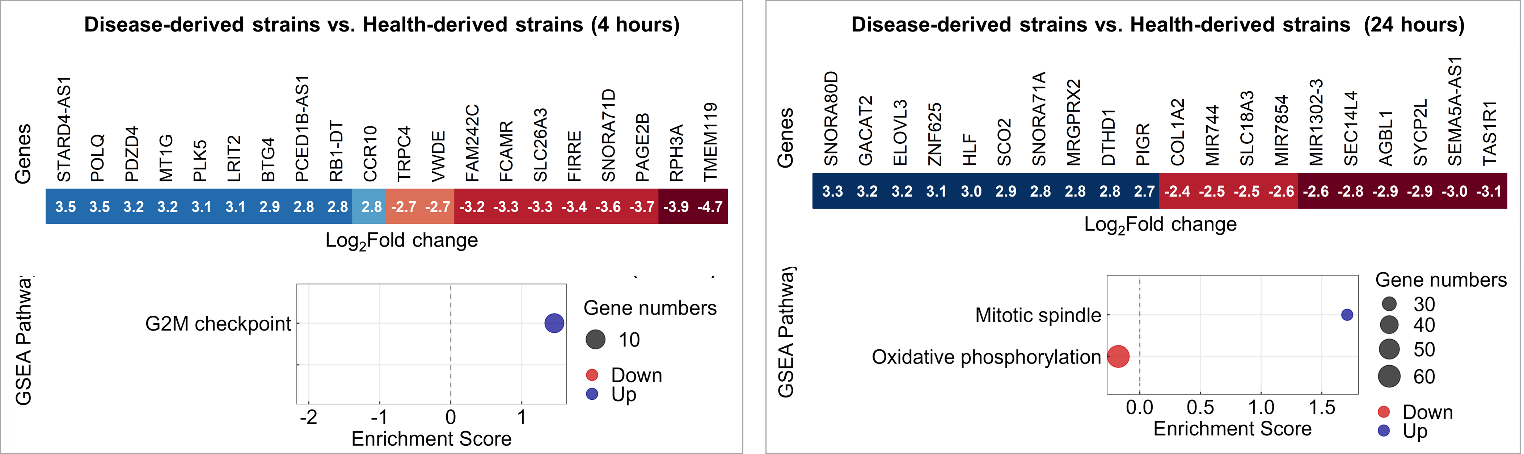


**Supplementary Figure 2. Transcriptional responses of DOK to infection with clinical isolates of *F. polymorphum* at 4 hours.**


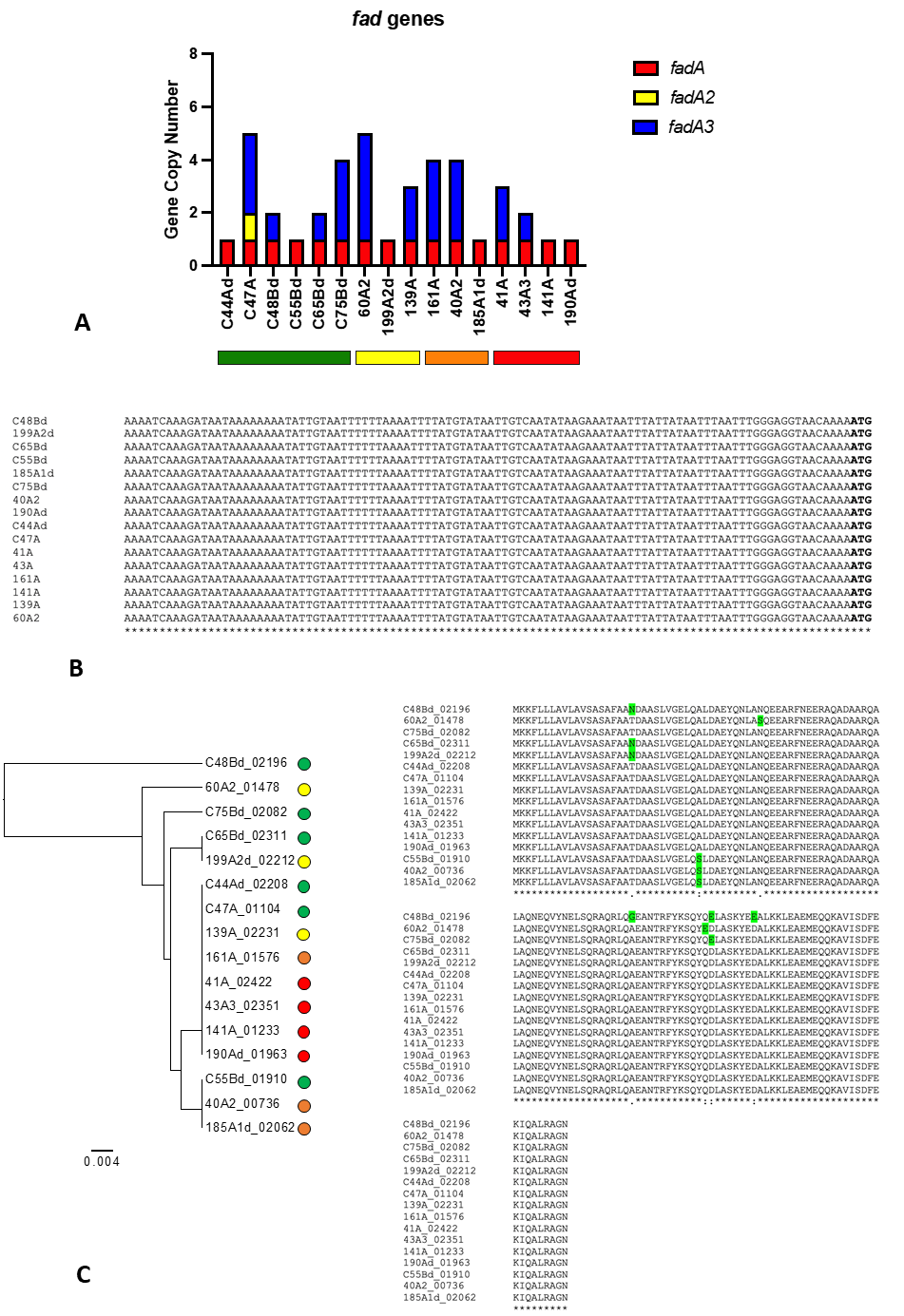


**Supplementary Figure 2. FadA gene analysis of 16 F. polymorphum isolates. A)** Gene copy number, B) promotor sequences, and C) gene sequences.
